# Supplementary figures and images for: Funneling modulatory peptide design with generative models: Discovery and characterization of disruptors of calcineurin protein-protein interactions
Source: PLoS Comput Biol. 2023 Feb 2;19(2):e1010874. doi: 10.1371/journal.pcbi.1010874 (PMC9928118; doi:10.1371/journal.pcbi.1010874)

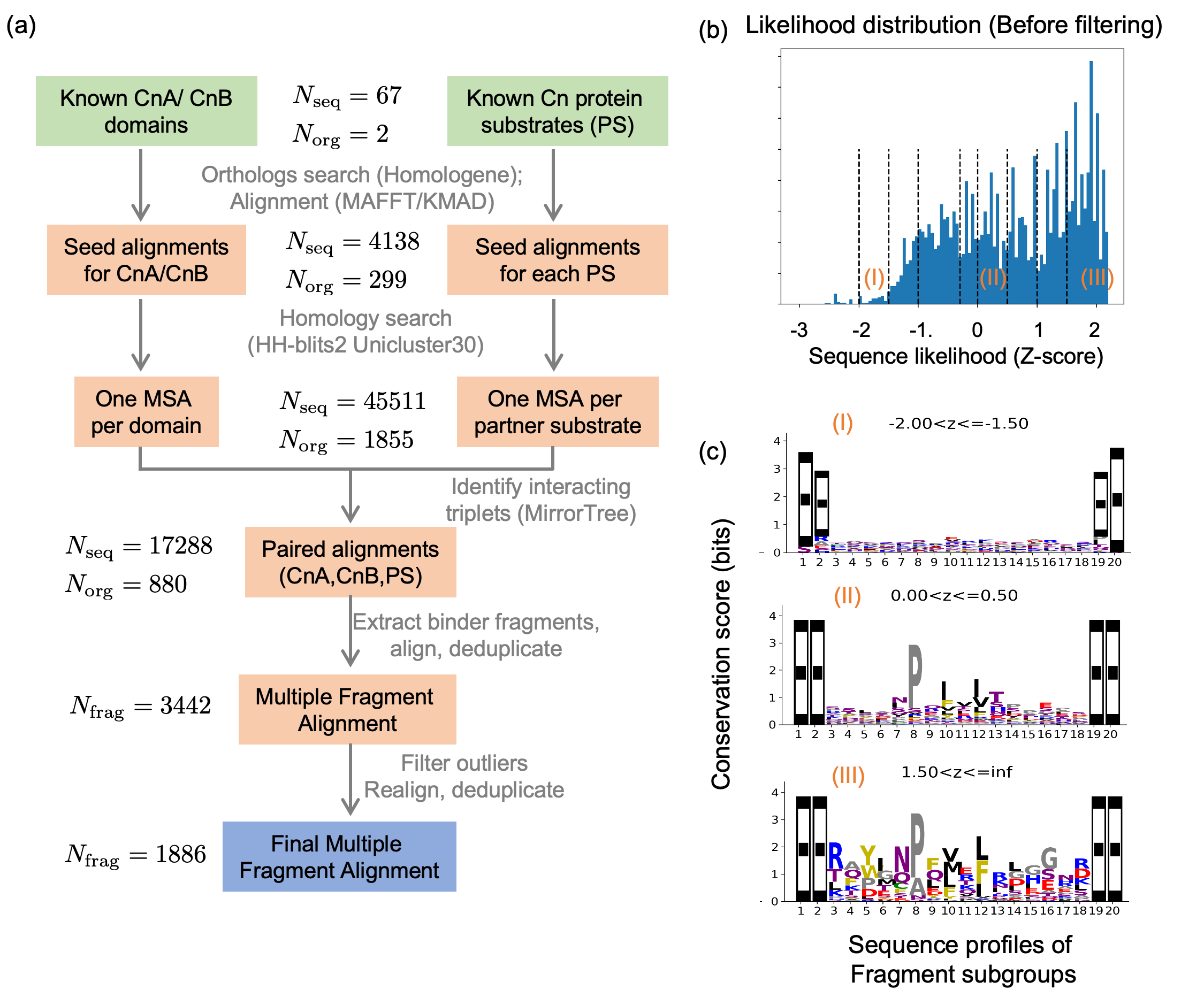

Supplement: S1 Fig — (a) Overview of the protocol for constructing the multiple fragment alignment. (b,c) cRBM-based refinement of the MFA obtained from the homology search: A cRBM model is trained on the MFA and likelihood scores are computed for all sequences (higher is better). Sequences with low likelihood values do not share the main conservation and coevolution patterns of the others and may be discarded. To determine a cut-off, we group sequences by likelihood, and visualize the sequence profile of each subgroup (c). Sequences with Z< -0.3 do not feature any conservation pattern, and are considered outliers. (PNG) [file pcbi.1010874.s002.png]

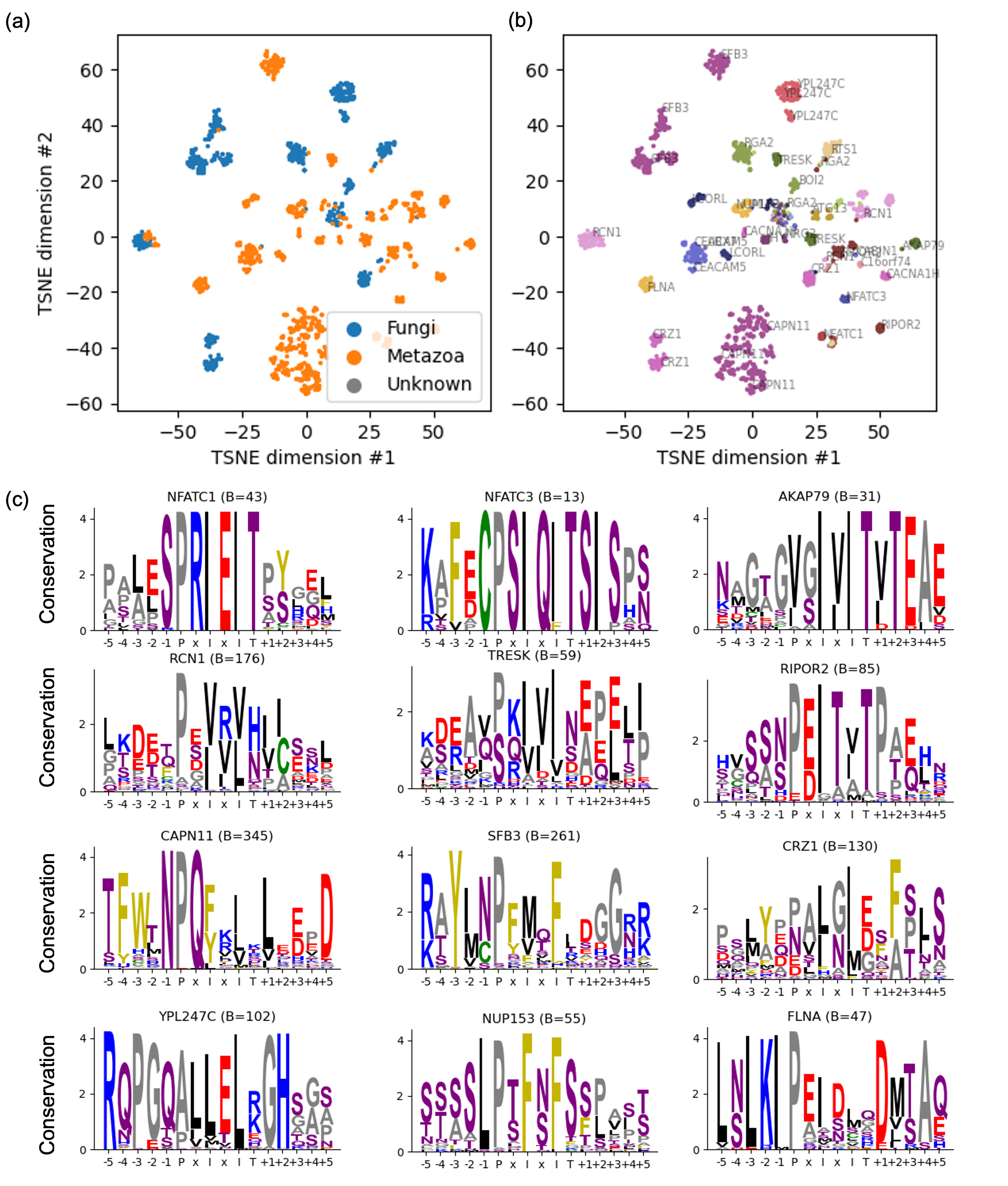

Supplement: S2 Fig — Selected data visualization of the multiple fragment alignment. (a,b) T-SNE visualization of the MFA, colored by phyla/gene reveals that fragments mainly cluster by gene. (c) Gene-specific sequence profiles, revealing a diversity of conserved binding motifs. B denotes the number of unique fragments found. (PNG) [file pcbi.1010874.s003.png]

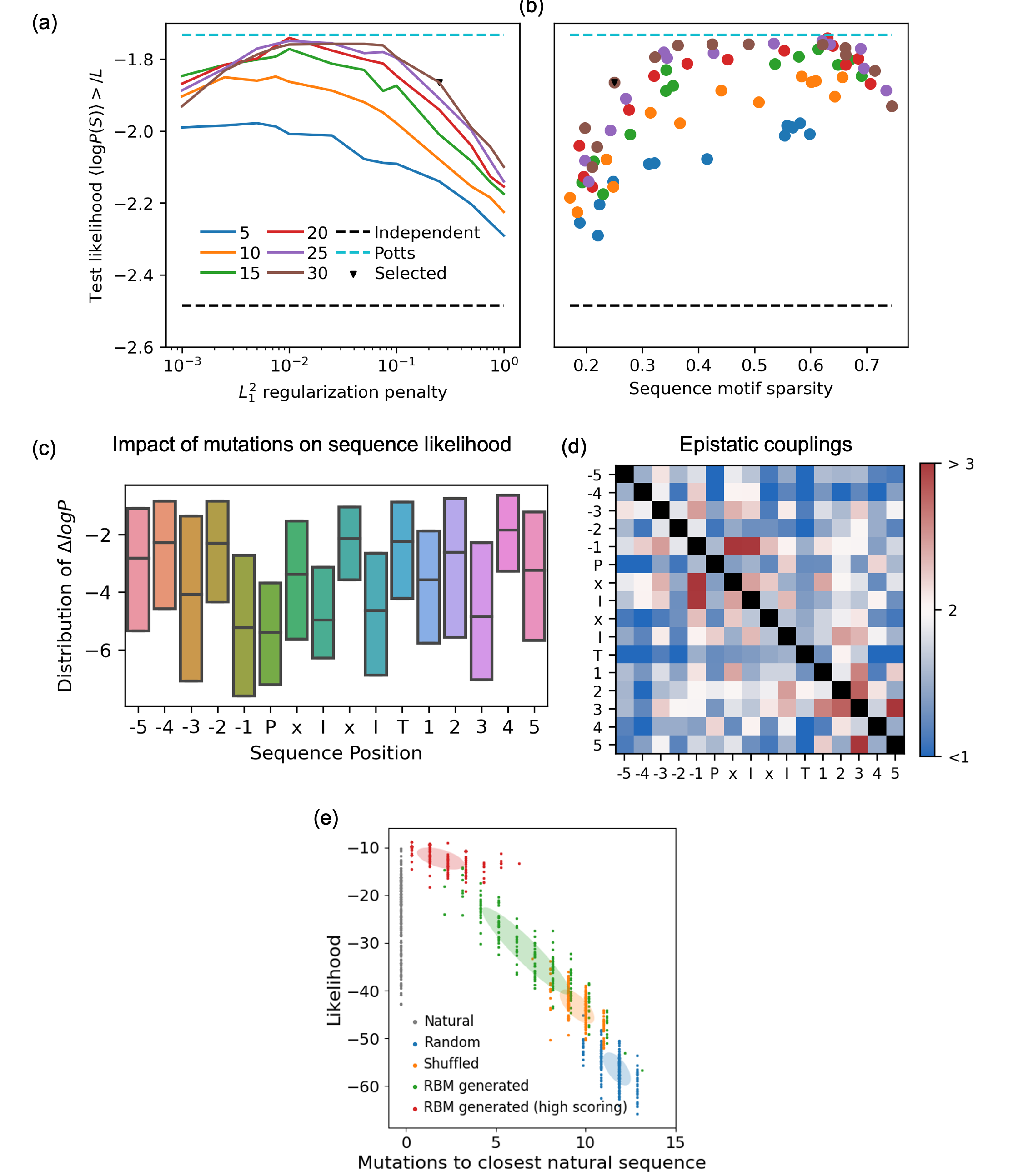

Supplement: S3 Fig — (a,b) Model selection protocol. A grid search is performed over the number of hidden units and values of sparse penalty. The model that achieves the best compromise between accuracy (high likelihood) and interpretability (low motif sparsity) is selected (black triangle). (c) Distribution of likelihood changes upon mutation grouped by position. The distribution is computed over all 19 possible mutations at each position for 100 representative fragments from the alignment (randomly selected by Kmeans++ algorithm). Positions with low average values are less tolerant to mutations and presumably more important for functionality. (d) Effective epistatic couplings learnt by the model, indicating significant covariation between core and flanking residues. (e) The quality-diversity trade-off of generated sequences. Scatter plot of sequence likelihood against number of mutations to the closest natural fragment. Sequences with likelihood similar or higher to the one of natural sequences but distinct from them can be generated. Conversely, random or PSSM-generated sequences are further away but distinguishable from natural fragments. (PNG) [file pcbi.1010874.s004.png]

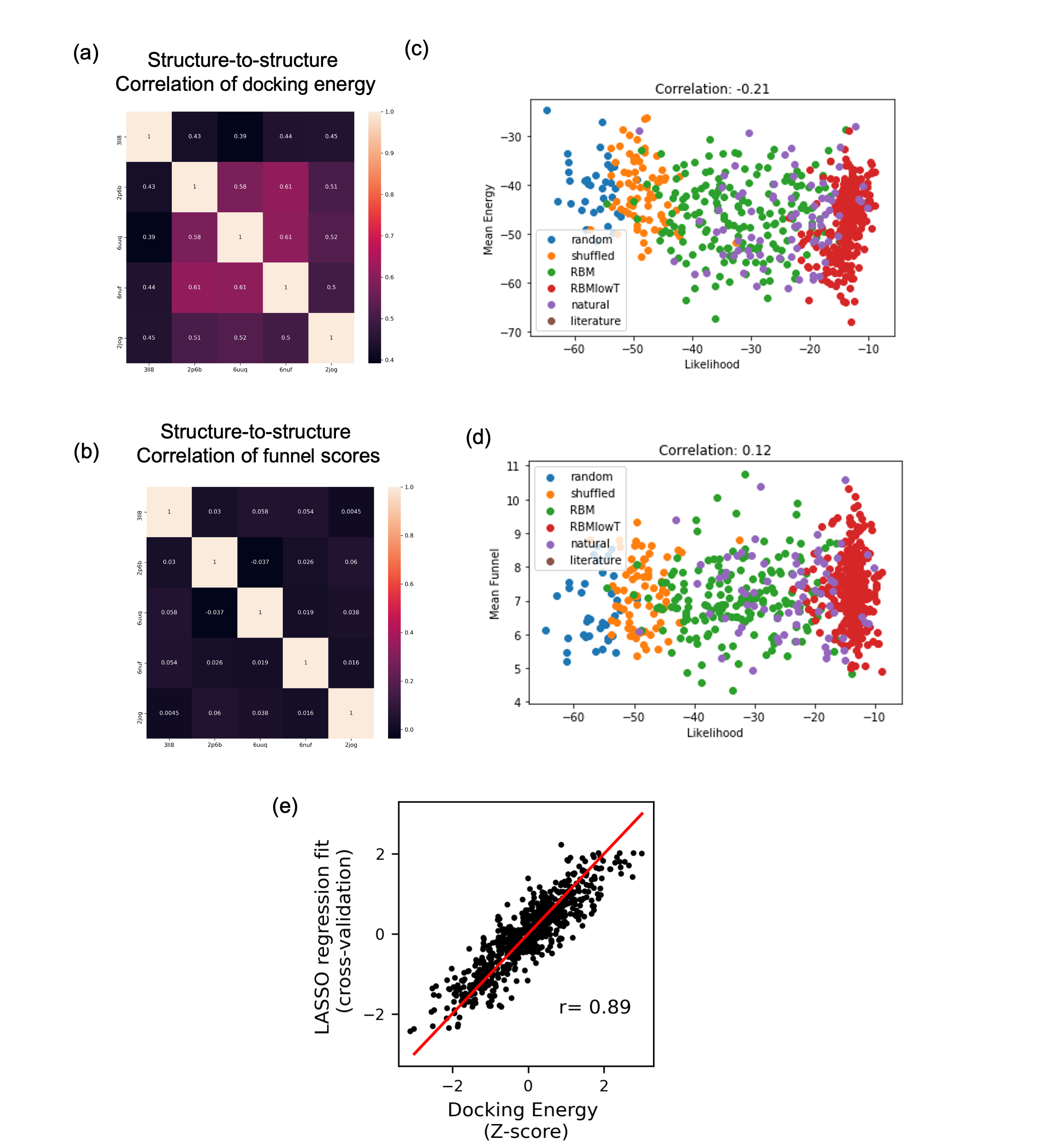

Supplement: S4 Fig — (a,b) Pearson cross-correlation matrix of the docking energy and funnel scores between five repeats using different crystal structures for Cn. (c,d) Scatter plot of the docking energy and funnel scores averaged over the five repeats against the sequence model likelihood; both are weakly but significantly correlated. (e) Single-site model fit of the docking energy scores by LASSO regression: scatter plot between docking energies and cross-validated predictions. (PNG) [file pcbi.1010874.s005.png]

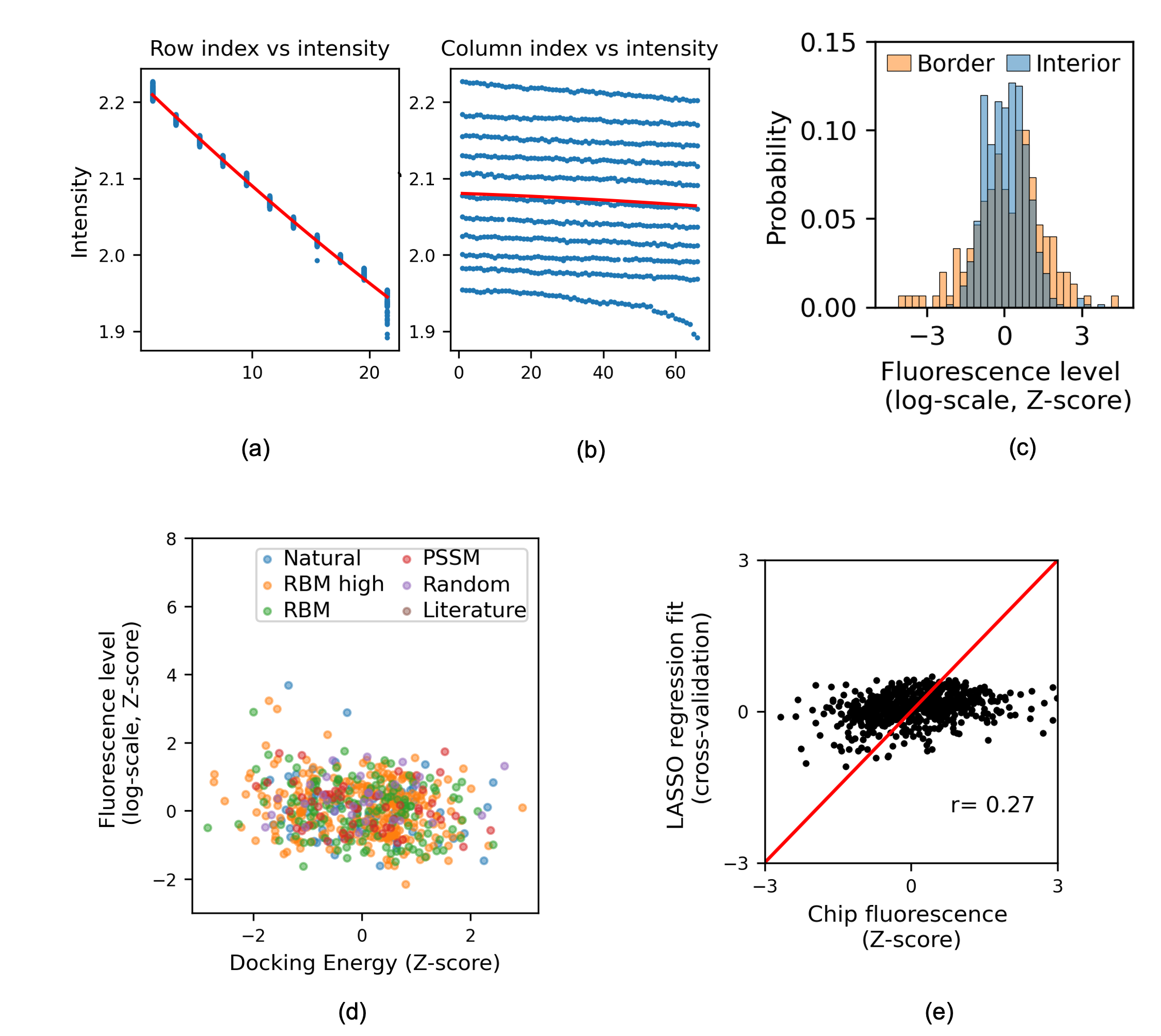

Supplement: S5 Fig — (a,b) Scatter plot of the fluorescence intensity level against the row and column index along the chip for one of the seven repeats (one point per peptide). A trend is fitted (red curve) and removed before further analysis. (c) Distribution of fluorescence intensity level (logarithmic scale, normalized to zero mean and unit variance, averaged over the seven repeats), for peptides printed at the border and at the interior of the chip. Spillover from the neighboring fluorescent tags results in higher fluorescence levels for border peptides; positive hits along the border were ignored in downstream analysis. (d) Scatter plot of the docking energy score against the fluorescence level. (e) Single-site model fit of the docking energy scores by LASSO regression: scatter plot between docking energies and cross-validated predictions. (PNG) [file pcbi.1010874.s006.png]

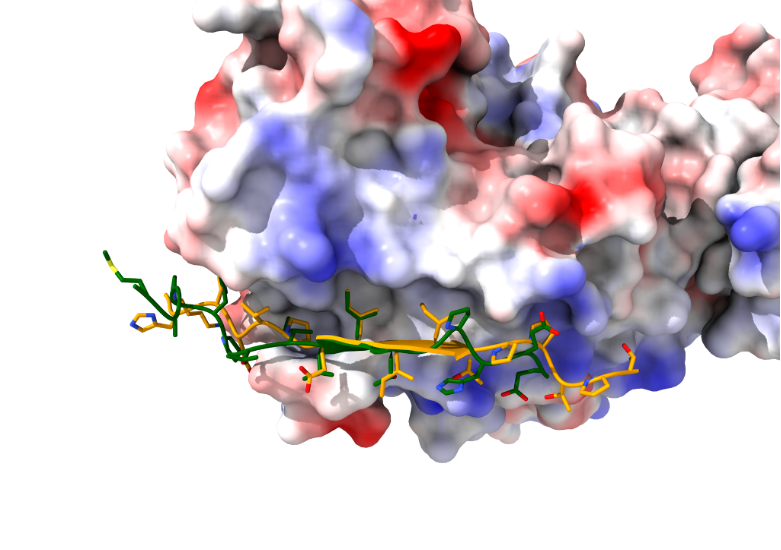

Supplement: S6 Fig — Calcineurin is shown in surface representation, colored by electrostatic potential. PVIVIT and C16orf74 peptides are shown in stick representation, respectively in green and orange. Visualization was performed using ChimeraX. (PNG) [file pcbi.1010874.s007.png]

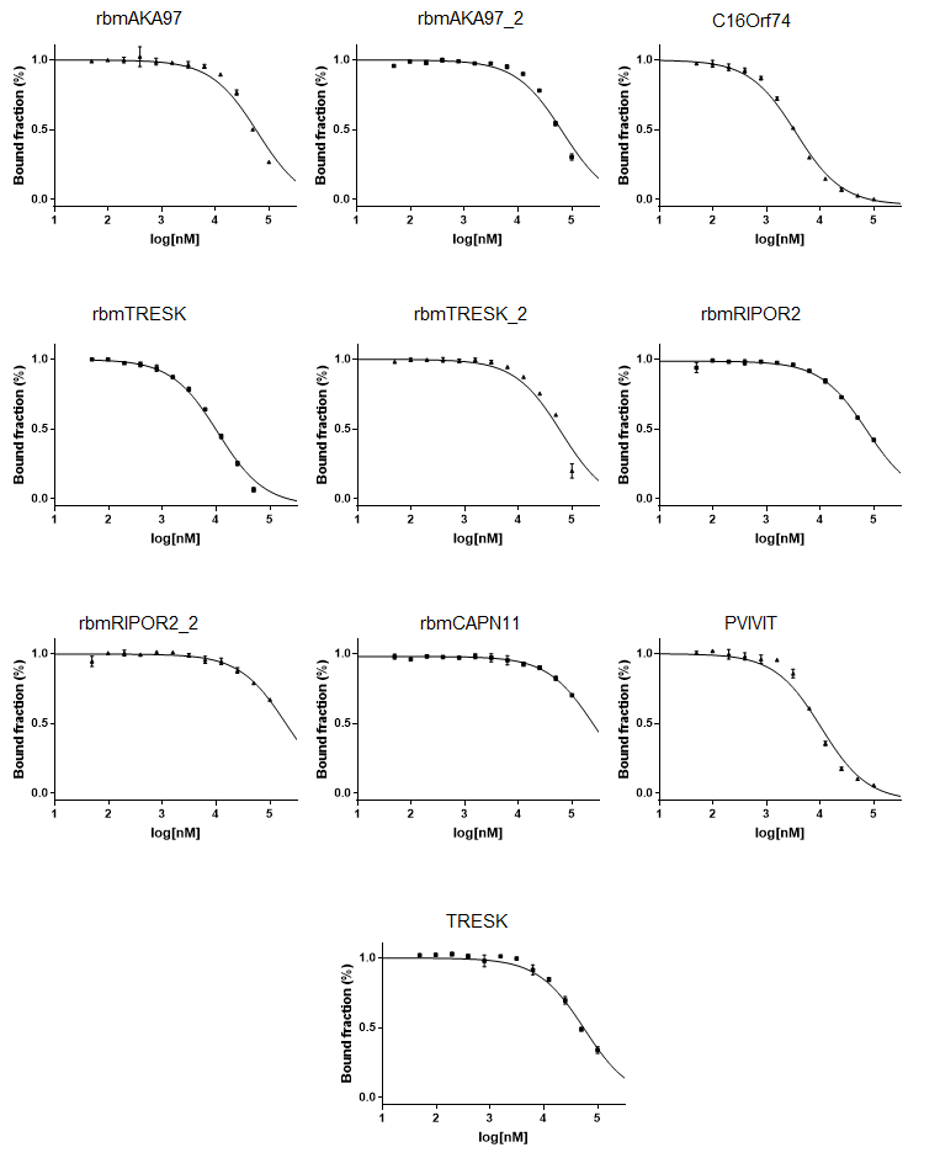

Supplement: S7 Fig — Variable concentrations of each of the peptides were incubated with the co-complex of Cn and PVIVIT. Polarization values were fitted to a single site inhibition model and IC50 values were extracted. (PNG) [file pcbi.1010874.s008.png]
