# Supplementary material for: Funneling modulatory peptide design with generative models: Discovery and characterization of disruptors of calcineurin protein-protein interactions
Source: PLoS Comput Biol. 2023 Feb 2;19(2):e1010874. doi: 10.1371/journal.pcbi.1010874 (PMC9928118; doi:10.1371/journal.pcbi.1010874)
Supplement: S4 Data — (PDF) [file pcbi.1010874.s012.pdf]

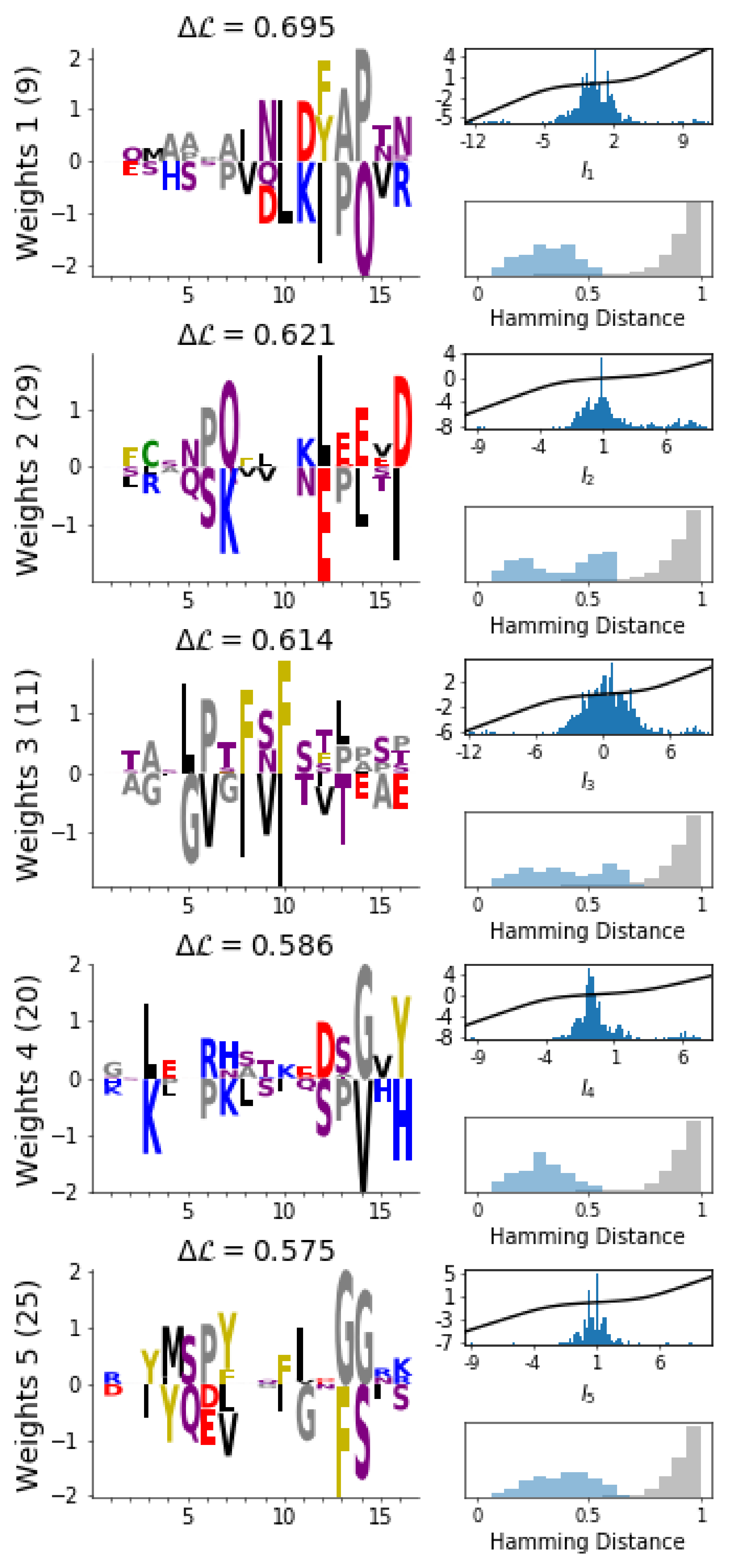

ΔL = 0.695
Weights 1 (9)
l1
Hamming Distance
ΔL = 0.621
Weights 2 (29)
l2
Hamming Distance
ΔL = 0.614
Weights 3 (11)
l3
Hamming Distance
ΔL = 0.586
Weights 4 (20)
l4
Hamming Distance
ΔL = 0.575
Weights 5 (25)
l5
Hamming Distance

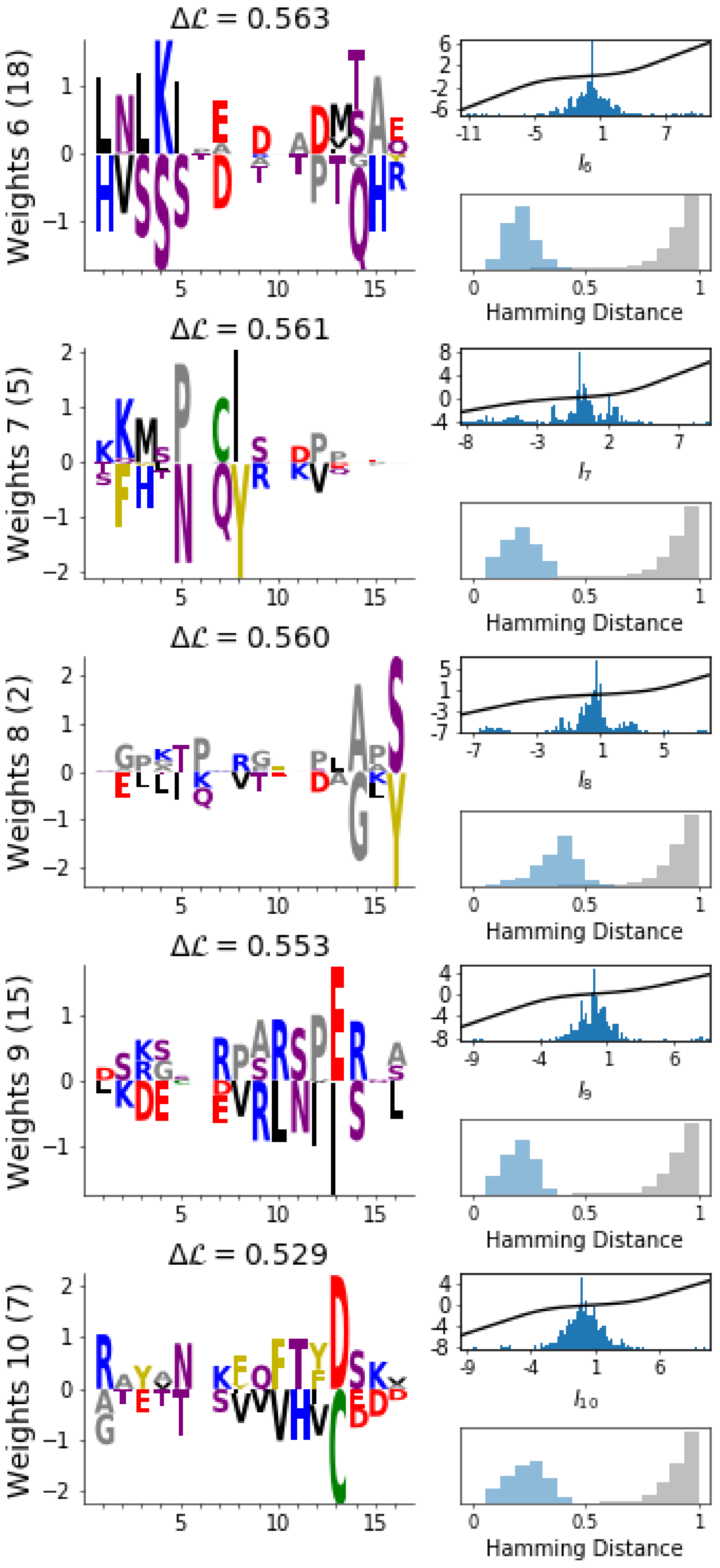

ΔL = 0.563
Weights 6 (18)
l6
Hamming Distance
ΔL = 0.561
Weights 7 (5)
l7
Hamming Distance
ΔL = 0.560
Weights 8 (2)
l8
Hamming Distance
ΔL = 0.553
Weights 9 (15)
l9
Hamming Distance
ΔL = 0.529
Weights 10 (7)
l10
Hamming Distance

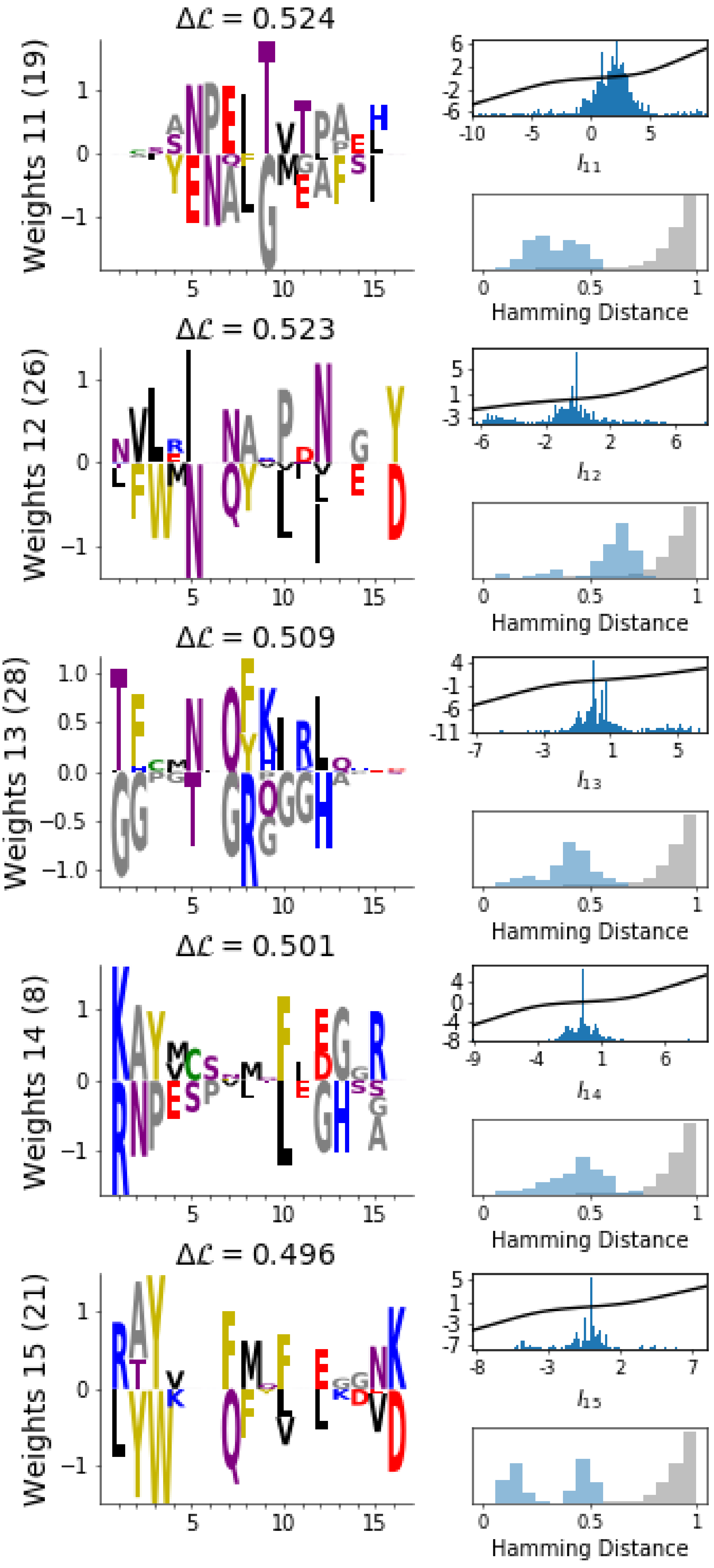

ΔL = 0.524
Weights 11 (19)
l11
Hamming Distance
ΔL = 0.523
Weights 12 (26)
l12
Hamming Distance
ΔL = 0.509
Weights 13 (28)
l13
Hamming Distance
ΔL = 0.501
Weights 14 (8)
l14
Hamming Distance
ΔL = 0.496
Weights 15 (21)
l15
Hamming Distance

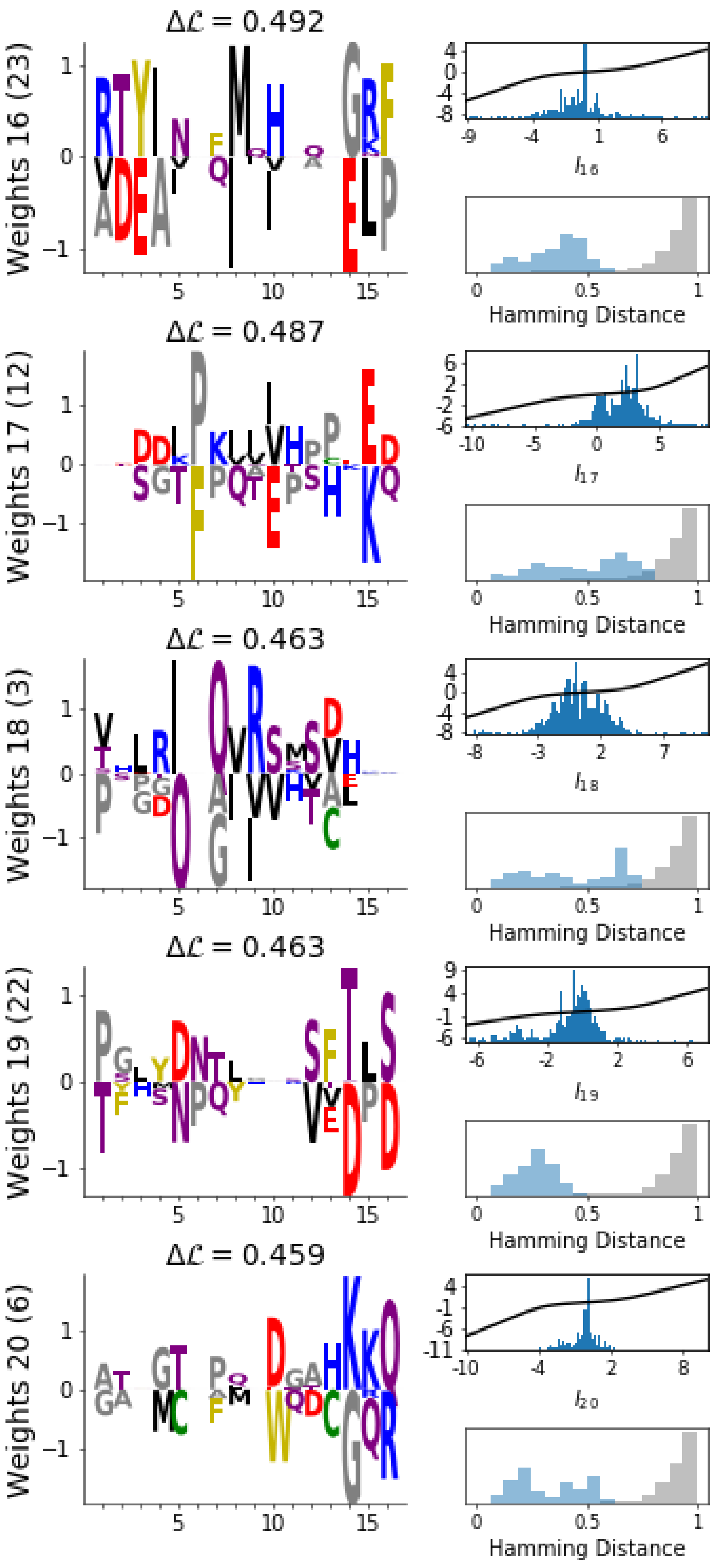
ΔL = 0.492
Weights 16 (23)
h_{16}
Hamming Distance
ΔL = 0.487
Weights 17 (12)
h_{17}
Hamming Distance
ΔL = 0.463
Weights 18 (3)
h_{18}
Hamming Distance
ΔL = 0.463
Weights 19 (22)
h_{19}
Hamming Distance
ΔL = 0.459
Weights 20 (6)
h_{20}
Hamming Distance

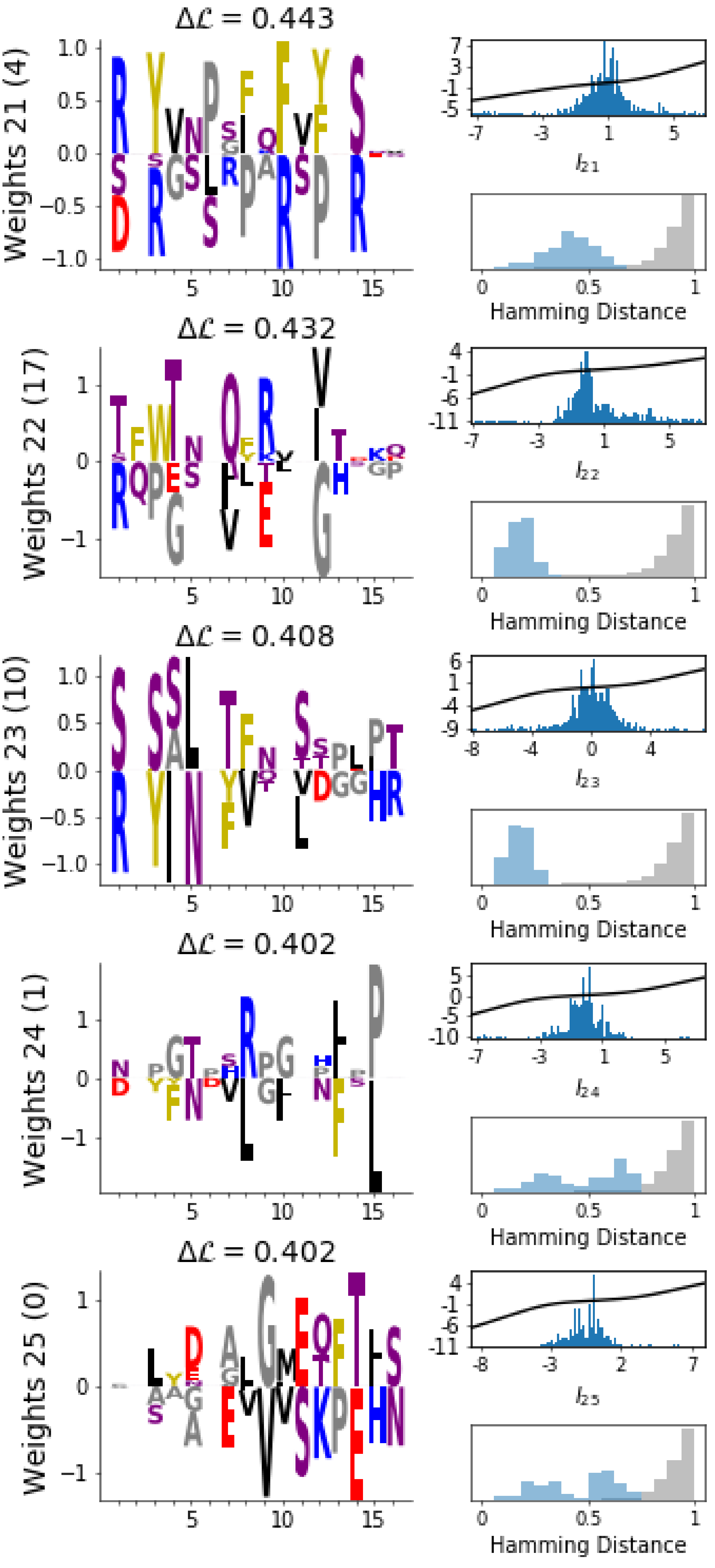

ΔL = 0.443
Weights 21 (4)
l_{21}
Hamming Distance
ΔL = 0.432
Weights 22 (17)
l_{22}
Hamming Distance
ΔL = 0.408
Weights 23 (10)
l_{23}
Hamming Distance
ΔL = 0.402
Weights 24 (1)
l_{24}
Hamming Distance
ΔL = 0.402
Weights 25 (0)
l_{25}
Hamming Distance

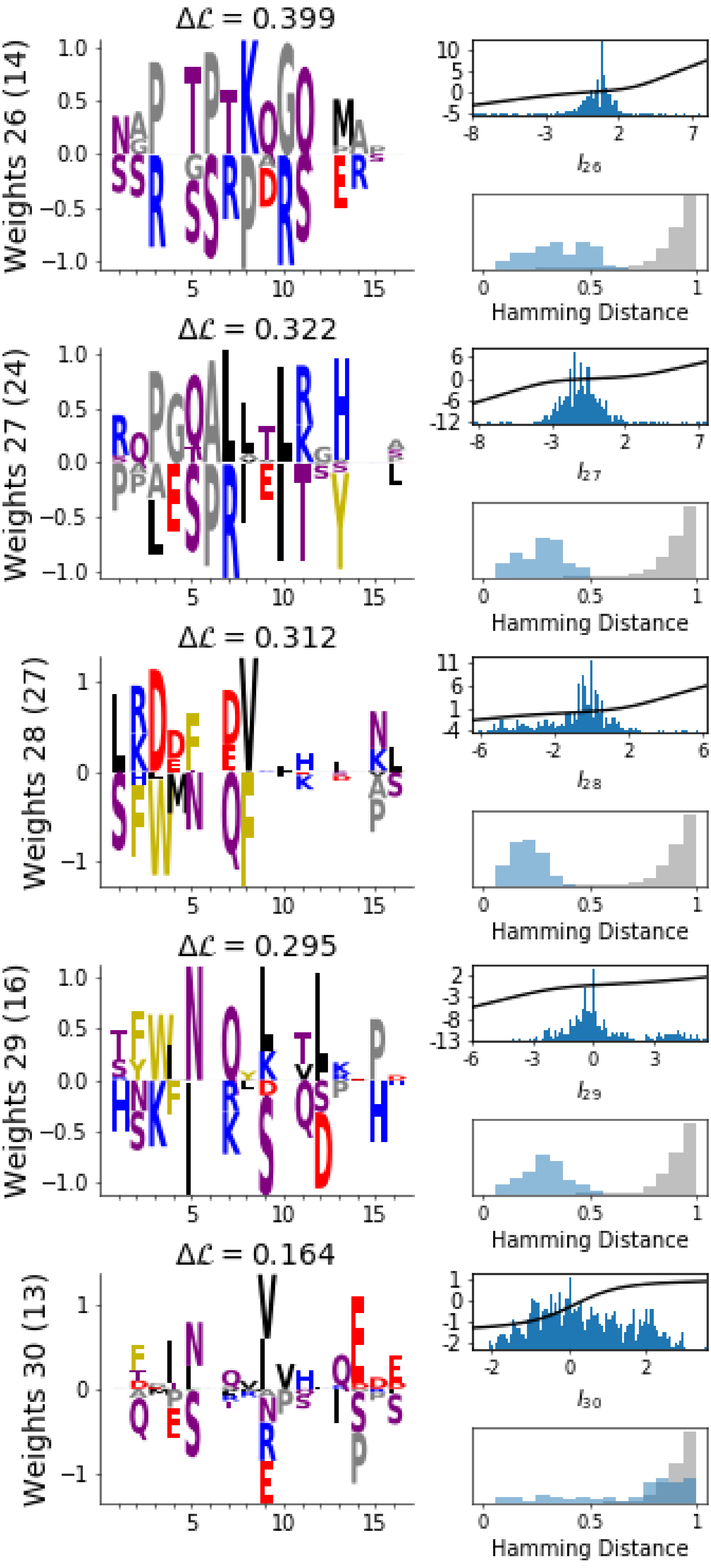

ΔL = 0.399
Weights 26 (14)
l26
Hamming Distance
ΔL = 0.322
Weights 27 (24)
l27
Hamming Distance
ΔL = 0.312
Weights 28 (27)
l28
Hamming Distance
ΔL = 0.295
Weights 29 (16)
l29
Hamming Distance
ΔL = 0.164
Weights 30 (13)
l30
Hamming Distance
