# Supplementary material for: Funneling modulatory peptide design with generative models: Discovery and characterization of disruptors of calcineurin protein-protein interactions
Source: PLoS Comput Biol. 2023 Feb 2;19(2):e1010874. doi: 10.1371/journal.pcbi.1010874 (PMC9928118; doi:10.1371/journal.pcbi.1010874)
Supplement: Attachment — Submitted filename: PCOMPBIOL-D-22-01508_reviewer.docx [file pcbi.1010874.s014.docx]

Dear Editor,

The paper titled “Funneling modulatory peptide design with generative models: discovery and characterization of disruptors of calcineurin protein-protein interactions” evaluates and designs the peptide binders for targeting “undruggable” protein-protein interfaces. This paper describes a peptide design protocol that harnesses this diversity via a machine learning generative model. As described in the paper, after identifying putative natural binding fragments by literature and homology search, a compositional Restricted Boltzmann Machine is trained and sampled to yield hundreds of diverse candidate peptides. The latter are further filtered via flexible molecular docking and an in-vitro microchip-based binding assay. Also, they have validated and tested the protocol on calcineurin, a calcium-dependent protein phosphatase involved in various cellular pathways in health and disease.

However, the manuscript in general is interesting and investigates an important problem from practical perspective, but there are some minor issues that should be addressed before this paper could be recommended for publication in the journal of PLOS Computational Biology. In addition, the paper is quite well-written and structured, however, there are some typos to be fixed. Therefore, I would recommend the authors to go through the whole paper to fix the typos.

The issues to be addressed are:

1) I would suggest to properly using the abbreviations of the terms that were widely used in the paper. The current format of the paper is very difficult to read.
2) The main question regarding the written paper is that why the authors didn’t try different machine learning techniques and didn’t compare between then because as you know there are a lot of machine learning techniques for sequence generative models.
3) It is very confusing how you have calculated probability formula P(S) in line 229 page 11.
4) The authors should explain why they have used LASSO regression and T-SNE dimensionality reduction for analysing data set. Why you have used other techniques for dimensionality reduction.
5) Use either English or American style throughout the paper.

In my conclusion the presented paper is written very well and can be accepted for publication with some miner revisions.
