# Supplementary material for: Funneling modulatory peptide design with generative models: Discovery and characterization of disruptors of calcineurin protein-protein interactions
Source: PLoS Comput Biol. 2023 Feb 2;19(2):e1010874. doi: 10.1371/journal.pcbi.1010874 (PMC9928118; doi:10.1371/journal.pcbi.1010874)
Supplement: Attachment — Submitted filename: answer_referees.docx [file pcbi.1010874.s015.docx]

Dear Pr. Papaleo, Pr. Elofsson and Dr. Martins,

We thank you and the anonymous reviewer for your consideration and the favorable comments. Please find below a point-by-point answer to the referee’s comments, and the updated manuscript attached. We look forward to seeing our article published at PLOS CB, and wish you happy holidays!

With best regards,

Dr. Jérôme Tubiana, on behalf of the authors

Reviewer #1: The authors introduce an interesting methodology for computer-aided SLiM design, making use of interesting machine learning methods and experimental validation of suggested targets. While some of the in vitro and in silico methods did not yield the expected results in regards to controls, this should not preclude the publication of this novel approach to small peptide design for protein-protein binding.

We thank the reviewer for finding our methodology novel and interesting. While we could not detect binding in the microarray experiment for several positive controls (known Calcineurin binders) – meaning that it has high false negative rate - it did help us identifying strong binders. Altogether, we are very happy with the presented methodology and its overall success rate (~70%).

As for revision requests, two minor issues stand out and, in my opinion, should be corrected before publication.

Line 246 - The trade-off between accuracy and interpretability, present in Supplementary figure 3(a,b) should be added to the main text and be expanded upon.

We thank the reviewer for pointing out this issue. We expanded this sentence as follows:

We trained multiple cRBMs onto the multiple fragment alignment by varying M and regularization strengths, and selected one that best compromised between performance and interpretability (Methods, Supplementary Figure 3a, 3b). Here, performance and interpretability of a model were respectively quantified by the log-likelihood of held-out test data (*i.e.* its ability to assign high probability to unseen natural binders) and the fraction of non-zero entries of (lower is more interpretable). Sparsity eases visualization and interpretation of the learnt sequence motifs, and stirs the generative model to a so-called compositional regime in which only a few motifs are present per sequence.

Despite our best efforts, we could not fit panels of Supplementary Figure 3a/b into Figure 3 (which is already full-page) without compromising readability.

Line 292-295 - Please be more explicit in regards to which positive and negative controls were selected and how they were selected, as it is especially important considering the lack of control correlation later observed in silico and in vitro.

We thank the reviewer for commenting on this point. We expanded this sentence as follows:

For comparison purpose, we further included: (i) *As positive controls*, two synthetic peptides (PVIVIT and PKIVIT) known to bind Cn at high affinity, (ii) *As negative controls*, 36 purely random 16-length peptides (iii) *Natural binding fragments* from the four human NFATc1-4 genes, plus 70 additional representatives (**Methods**) of the MFA; the majority of these are expected to bind Cn. (iv) As a baseline generative model, 72 samples from the PSSM model.

Reviewer #2:

The paper titled “Funneling modulatory peptide design with generative models: discovery and characterization of disruptors of calcineurin protein-protein interactions” evaluates and designs the peptide binders for targeting “undruggable” protein-protein interfaces. This paper describes a peptide design protocol that harnesses this diversity via a machine learning generative model. As described in the paper, after identifying putative natural binding fragments by literature and homology search, a compositional Restricted Boltzmann Machine is trained and sampled to yield hundreds of diverse candidate peptides. The latter are further filtered via flexible molecular docking and an in-vitro microchip-based binding assay. Also, they have validated and tested the protocol on calcineurin, a calcium-dependent protein phosphatase involved in various cellular pathways in health and disease.

However, the manuscript in general is interesting and investigates an important problem from practical perspective, but there are some minor issues that should be addressed before this paper could be recommended for publication in the journal of PLOS Computational Biology. In addition, the paper is quite well-written and structured, however, there are some typos to be fixed. Therefore, I would recommend the authors to go through the whole paper to fix the typos.

We thank the reviewer for finding our methodology interesting and important and our article well-written, and thank him/her for pointing out the issues.

The issues to be addressed are:

1) I would suggest to properly using the abbreviations of the terms that were widely used in the paper. The current format of the paper is very difficult to read.

Assuming that the reviewer meant “to properly define the abbreviations”, we have updated the manuscript to reduce abbreviation usage and redefine them multiple times throughout the manuscript.

2) The main question regarding the written paper is that why the authors didn’t try different machine learning techniques and didn’t compare between then because as you know there are a lot of machine learning techniques for sequence generative models.

We agree with the reviewer that they are many possible choices for the sequence generative models, such as Potts models, Variational Autoencoders (VAE), autoregressive 1D CNNs, RNNs or transformers (see review by Wu et al. COCB 2021), and that some could be suitable as well. However, **there is currently no established gold standard method** for MSA-based generative modeling of protein sequences, and a thorough comparison of the aforementioned methods is outside of the scope of this paper. Therefore, we only compared cRBMs to PSSMs, the gold standard for evolutionary-based design prior to SGMs, and found that cRBMs outperformed PSSMs (evidence summarized below). We also found that Potts model modeled well the natural fragment distribution, suggesting that they would be suitable as well.

In the revised submission, we also added a comparison of the cRBM with ProteinMPNN, a recently published **structure-based** sequence generative model on the DDG task based on Nguyen et al. eLife 2019, and found that cRBM outperformed it on three of the four mutagenesis datasets, and performed equivalently on the last one. The following paragraphs were added to the manuscript:

*Results section:* The cRBM-based<https://www.codecogs.com/eqnedit.php?latex=%20%5CDelta%20%5Clog%20P#0> also correlated better with<https://www.codecogs.com/eqnedit.php?latex=%5CDelta%20%5CDelta%20G#0> measurements than log-likelihood differences computed using ProteinMPNN (1), a recently published graph neural network for structure-based protein design (, reference complex:2p6b:AB-E).

*Discussion:* How robust is the protocol with respect to the choice of the evolutionary-based sequence generative model? Here, we used compositional Restricted Boltzmann Machines (cRBM), an expressive generative model with a closed-form likelihood function and tractable number of parameters that generalizes over PSSM (i.e., independent model) and Potts models by incorporating sparse, high-order interactions. A protocol solely based on PSSMs, which neglects inter-dependencies between positions would yield a much lower success rate, as evidenced by (i) entropy calculations, which show that explicitly modeling inter-dependencies reduces the size of the space of candidate sequences by a factor of at least ~4000 (Methods) (ii) flexible docking simulations, which show that PSSM-generated sequences have worse docking energy than cRBM-generated ones on average and (iii) the microarray experiment, which did not identify any PSSM-generated sequence with high binding affinity to Cn. On the other hand, Potts models, which also integrate inter-dependencies, model equally well the distribution of natural binders (Methods, Supplementary Figure 3a), and would likely perform comparably. Other machine learning generative models that account for inter-dependencies (see Wu. et al. (1) for an overview) may be suitable as well. On the other hand, it is unclear whether large protein language models would perform equally well, as they are usually trained and evaluated on full-length sequences rather than on fragments; here, they may lack key contextual information (e.g., that the fragments belong to disordered regions).

*Discussion:* Accordingly, we found that our cRBM model better predicted binding affinity changes upon mutation that ProteinMPNN, a recently published structure-based autoregressive graph neural network for sequence design

*Supplementary section:* Added entropy values for the PSSM distribution (regular and low-temperature sampling); Added Spearman correlation coefficient for DDG prediction with ProteinMPNN.

3) It is very confusing how you have calculated probability formula P(S) in line 229 page 11.

Thank you for pointing out this issue. We updated Figure 3b and its caption to better fit this presentation of cRBMs. Starting from the classical formulation of RBMs (undirected graphical models on bipartite graphs), Eqn (1) is obtained by marginalizing over hidden units (see Tubiana, Cocco, Monasson eLife 2019).

New Figure 3b panel & caption:

Graphical depiction of the cRBM model, the parametric form chosen. The visible layer corresponds to the aligned sequence; each visible unit contributes a site-specific term to the log-likelihood. The hidden (representation) layer corresponds to unobserved hidden units, each of which contributes an additional term to the log-likelihood function, defined as a linear projection through a sparse tensor followed by a trainable, strictly convex non-linearity.

New sentence:

Equation (1) is derived from the classical RBM definition (i.e. an undirected graphical model with pairwise, bipartite interactions) by marginalizing the joint distribution over the hidden units (1).

4) The authors should explain why they have used LASSO regression and T-SNE dimensionality reduction for analysing data set. Why you have used other techniques for dimensionality reduction.

LASSO regression and T-SNE were used exclusively for data visualization purposes. Specifically, we used LASSO regression to fit a single-site model that best reproduced the docking experiment, and a 2D T-SNE projection to visualize the distribution of fragments and reveal the presence of clusters.

Updated Methods paragraph:

All statistical analysis, including statistical tests, LASSO regression (Used in Figure 4c Supplementary Figure 5e for fitting single-site models to the results of the molecular docking and microarray binding experiment) and T-SNE dimensionality reduction (for visualizing the distribution of natural fragments and highlighting clusters in Supplementary Figure 2a,b) were performed using the numpy, scipy and scikit-learn Python packages.

5) Use either English or American style throughout the paper.

Corrected wherever found

In my conclusion the presented paper is written very well and can be accepted for publication with some miner revisions.

Other changes: Supplementary Table 1 was missing from the Supplementary Material and is now included.
